# Electrochemical Immunosensor for the Determination of Antibodies against Prostate-Specific Antigen Based on ZnO Nanostructures

**DOI:** 10.3390/ijms24065803

**Published:** 2023-03-18

**Authors:** Viktorija Liustrovaite, Dovydas Karoblis, Benediktas Brasiunas, Anton Popov, Arturas Katelnikovas, Aivaras Kareiva, Arunas Ramanavicius, Roman Viter, Maria Teresa Giardi, Donats Erts, Almira Ramanaviciene

**Affiliations:** 1 NanoTechnas—Center of Nanotechnology and Materials Science, Institute of Chemistry, Faculty of Chemistry and Geosciences, Vilnius University, Naugarduko St. 24, LT-03225 Vilnius, Lithuania; 2 Institute of Chemistry, Vilnius University, Naugarduko St. 24, LT-03225 Vilnius, Lithuania; 3 Institute of Atomic Physics and Spectroscopy, University of Latvia, Jelgavas St. 3, LV-1004 Riga, Latvia; 4 Institute of Crystallography, National Research Council, AdR1, 00010 Montelibretti, Italy; 5 Biosensor Srl, Via Degli Olmetti 44, Formello, 00060 Rome, Italy; 6 Institute of Chemical Physics, University of Latvia, 19 Raina Blvd., LV-1586 Riga, Latvia

**Keywords:** zinc oxide nanostructures, one-dimensional nanostructures, electrochemical immunosensor, cyclic voltammetry, differential pulse voltammetry, antigen–antibody complex

## Abstract

In this study, ZnO nanostructures with different types of morphologies and particle sizes were evaluated and applied for the development of an immunosensor. The first material was composed of spherical, polydisperse nanostructures with a particle size in the range of 10–160 nm. The second was made up of more compact rod-like spherical nanostructures with the diameter of these rods in the range of 50–400 nm, and approximately 98% of the particles were in the range of 20–70 nm. The last sample of ZnO was made up of rod-shaped particles with a diameter of 10–80 nm. These ZnO nanostructures were mixed with Nafion solution and drop-casted onto screen-printed carbon electrodes (SPCE), followed by a further immobilization of the prostate-specific antigen (PSA). The affinity interaction of PSA with monoclonal antibodies against PSA (anti-PSA) was evaluated using the differential pulse voltammetry technique. The limit of detection and limit of quantification of anti-PSA were determined as 1.35 nM and 4.08 nM for compact rod-shaped spherical ZnO nanostructures, and 2.36 nM and 7.15 nM for rod-shaped ZnO nanostructures, respectively.

## 1. Introduction

Semiconducting metal oxides have been extensively researched over the past few decades [1,2]. This type of material has strong ionic bonding between the positively charged metal and negatively charged oxygen ions. They exhibit various properties, including a high surface area [3], good thermal and chemical stability [3,4], wide bandgap (>2 eV) [5], high dielectric constant [6], ultrahigh carrier mobility, and superior oxidation resistance [7]. Moreover, many semiconducting metal oxides consist of abundant elements found in the Earth’s crust, making them inexpensive and environmentally friendly. These characteristics make metal oxides applicable in various fields, ranging from sensors for numerous gas detection [8] to light-emitting diodes [9], electrochromic devices [10], or immunosensor design [11]. Metallic nanomaterials can also be employed to improve biomarker detection characteristics [12].

Among numerous semiconducting metal oxides [2,13,14], one-dimensional ZnO nanostructures are considered the most functional [15,16]. ZnO nanostructures have been used in active antibacterial food packaging [17], photoelectrochemical water splitting [18], low-operating temperature sensors for NO_2_ detection [19], self-powered UV photodetectors [20], supercapacitor electrodes [21], etc. There are many different synthesis methods for the preparation of one-dimensional ZnO nanostructures. This material can be synthesized with different morphologies, including nanorods [22], nanotubes [23], nanospheres [24], nanotetrapods [25], nanowires [26], nanobelts [27], nanobullets [28], etc. Many studies have been performed to investigate how the size and shape of ZnO nanoparticles influence optical [29], electrical [30], or electrochemical [31] properties. Furthermore, different ZnO morphologies influence the sensing performance of electrochemical [32] or photoluminescence-based biosensors [33]. Biosensors based on ZnO nanostructures can offer a high sensitivity, selectivity, rapid and precise response, and can be applied as an inexpensive alternative for cancer detection [34].

Despite the large number of studies investigating the biosensors based on one-dimensional ZnO nanostructure matrix used to detect the enzymes associated with a significant number of diseases, there are only several publications describing the ZnO-based detection of cancer biomarkers and cells [35,36]. Therefore, the development of point-of-care immunosensor devices for the early detection of cancer can result in novel ways of treating malignancies and improving patient care through real-time monitoring. However, to achieve the abovementioned goals, some specifications must be met, namely, a quick label-free and selective identification of cancer markers, small sensor size, affordability, and portability. Therefore, to meet these specifications, we used carbon electrodes that were produced using screen printing technique in a repeatable, inexpensive, and disposable way [37]. SPCE are suitable for various fields of electrochemistry and particularly for chemical and biological sensing [38].

PSA, which is also known as kallikrein-related peptidase 3, is one of the most common proteins secreted by the normal human prostate epithelium and seminal plasma. PSA is an androgen-dependent glycoprotein of 30 kDa with chymotrypsin-like enzymatic activity that is important in the fragmentation of seminal vesicle-released proteins (semenogelins) [39]. PSA has established itself as the most accurate clinical biomarker for diagnosing prostate cancer, which is the leading cause of death in men between the ages of 55 and 80. As of now, the disease cannot be cured once the disease spreads [40]. As such, there is an urgent necessity for developing quick and precise tests to facilitate the early detection, limit the spread, and track the course of the disease. For this purpose, immunosensors can be used to detect PSA, the overexpression of which is an indicator of prostate cancer. On the other hand, the presence of autoantibodies against PSA (autoanti-PSA) in human serum can be measured. Research suggests that the generation of autoanti-PSA is related to prostate changes, specifically the enlargement of the prostate, called benign prostatic hyperplasia [41,42]. However, to the best of our knowledge, there is no reported research on electrochemical immunosensors based on ZnO nanostructures for the detection of anti-PSA.

In this work, the structural, morphological, and photoluminescent features of ZnO nanostructures were investigated and compared. Additionally, a novel electrochemical immunosensor was developed using SPCE modified with the selected ZnO nanostructures and PSA for anti-PSA detection. The efficiency of the developed electrochemical immunosensor was evaluated. Differential pulse voltammetry (DPV) and cyclic voltammetry (CV) methods were applied for the monitoring of the immunosensor response generated in the presence of a [Fe(CN)_6_]^3−/4−^-based redox probe.

## 2. Results and Discussion

**Figure 1:**
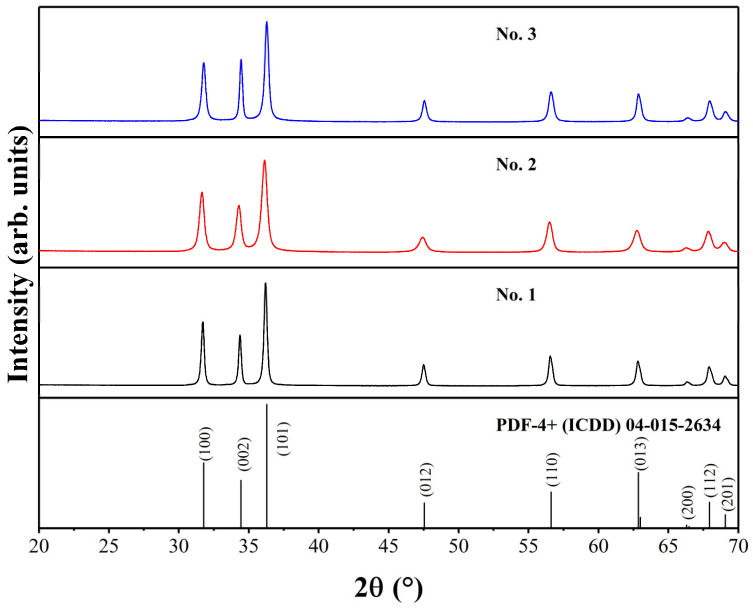
XRD patterns of ZnO samples.

For all ZnO samples, XRD analysis was performed to investigate the phase purity, crystal structure, and crystallite size. The XRD patterns of ZnO powders are presented in Figure 1. The formation of phase-pure products was observed regardless of different synthesis conditions. It is known that ZnO can have three possible crystal structures: hexagonal wurtzite, cubic zinc-blende, or cubic rocksalt type, which is only stable at high pressure [43].

In our case, the XRD profiles match very well with the hexagonal ZnO standard XRD data (PDF-4+ (ICDD) 04-015-2634; space group P6_3_mc, a = 3.25330 Å, c = 5.20730 Å). The broadest peaks can be seen for the No. 2 sample, which has a crystallite size of 18 nm. Other samples possess larger crystallites, with 24 nm for the No. 3 sample and 30 nm for the No. 1 sample. Rietveld refinement was performed for all ZnO compounds to investigate the unit cell parameters, and the results are presented in Table 1. The acquired unit cell parameters are very close to the parameters for the ZnO standard. The ideal ratio between two cell parameters (a/c) is considered to be 1.633 for hexagonal wurtzite unit cells [44]. For our samples, this ratio is 1.602 or 1.603, which is about 2% lower than a theoretical one.

**Figure 2:**
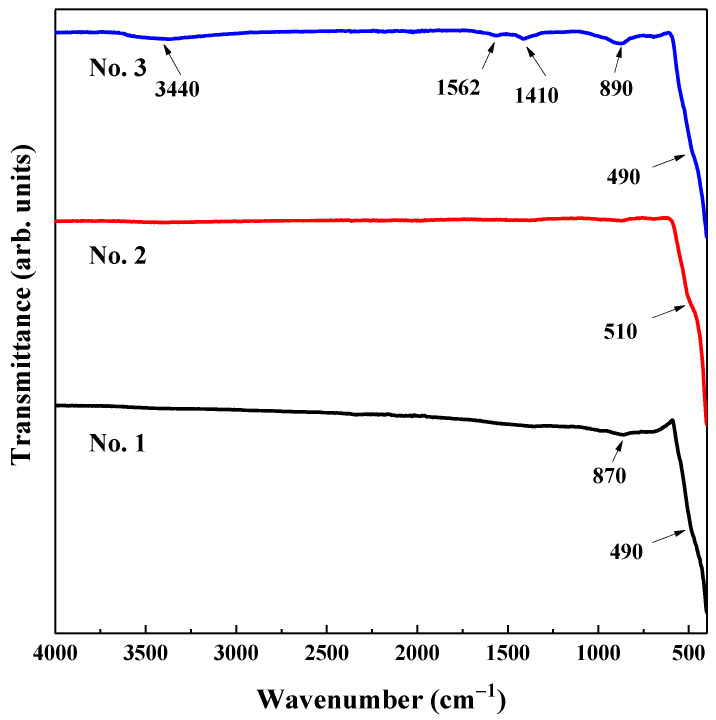
FT-IR spectra of ZnO samples.

The FT-IR spectra of all ZnO samples were recorded at room temperature in the 4000 to 400 cm^−1^ range and are represented in Figure 2. All samples show an absorption band in the 490–510 cm^−1^ interval, which can be associated with the stretching mode of the Zn-O bond. Moreover, No. 1 and No. 3 compounds contain low-intensity broadband at 870–890 cm^−1^, which could be related to tetrahedral-coordinated Zn [45].

Since ZnO materials were prepared at different temperatures, this led to some spectral changes, which resulted in additional bands for No. 3. One broad band centered around 3440 cm^−1^ could arise due to O-H bond stretching vibration in adsorbed water. The bands at 1562 and 1410 cm^−1^ can be linked to the asymmetric and symmetric stretching vibrations of the carboxylic group, which could originate from a small residue of zinc acetate used in the synthesis of this material. Moreover, the low synthesis and drying temperature could influence the small amounts of acetate groups found for the No. 3 compound.

**Figure 3:**
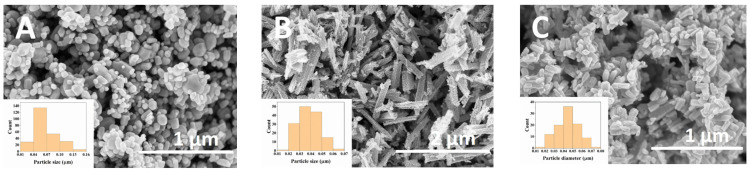
SEM images of No. 1 (**A**), No. 2 (**B**), and No. 3 (**C**) ZnO samples.

Figure 3 displays the SEM images of different ZnO samples. It can be seen that all the compounds contain different morphologies with varying particle sizes. No. 1 material (Figure 3A) is composed of polydisperse round-shape nanostructures with a particle size in the 10–160 nm region. The No. 2 ZnO (Figure 3B) sample is composed of smaller spherical nanostructures, which are collated into rods. Around 98% of the particles are in the 20–70 nm range, while the diameter of these rods is in the 50–400 nm interval. The length of the rods varies from 300 nm up to 1.8 µm. Last, the ZnO No. 3 (Figure 3C) sample consists of rod-like particles with a diameter of 10–80 nm. The length of these rods is in the 50–200 nm interval, which is smaller in comparison to the No. 2 sample. Moreover, this sample is not composed of smaller nanostructures, which was the case for the No. 2 sample. It should be noted that the morphology observed in two of our samples (No. 1 and No. 2) is significantly different than in previously reported studies with the same synthesis conditions [46,47]. Flake-like [46] and round-shape [47] particles were obtained in previous works. The No. 3 sample displayed a similar rod-like morphology, which is in good agreement with previously reported ZnO [48]. The reproducibility of similar-shaped nanoparticles is considered to be a difficult task, where even minor changes in the experimental parameters or the commercial source of the starting materials can lead to batch-to-batch variations [49].

**Figure 4:**
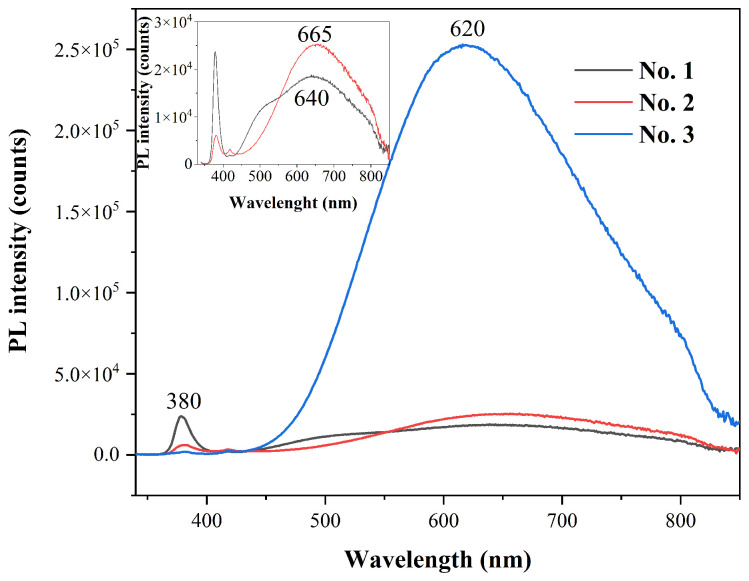
Photoluminescence spectra (λ_ex_ = 325 nm) of different ZnO samples. The inset represents the magnified view of No. 1 and No. 2 photoluminescence spectra.

Different morphologies of the ZnO nanostructures can influence the optical properties; therefore, photoluminescence measurements were performed. The emission spectra for all ZnO samples are demonstrated in Figure 4. It is known that the emission spectra of undoped ZnO contains two of the most prominent emission bands: a near-band edge in the UV region and a deep-level emission in the visible light region [50]. In our case, three emission bands can be observed: one centered around 380 nm, the second one at 420 nm, and the last one at 620, 640, and 665 nm, respectively. The emission detected at 380 nm is associated with the recombination of free excitons between the conductive band and the valence band [51]. The intensity of this band for ZnO No. 1 is larger than the emission band in the visible region, which could be related to the high crystallinity of this sample [52], which correlates with XRD data. Moreover, a low-intensity band at 420 nm can arise due to interstitial zinc defect in the ZnO structure, which was previously observed for ZnO tetrapods [53]. In addition, a broad red emission band found at 620–665 nm can be linked to interstitial oxygen ions [54]. The intensity ratio between the visible peak and UV peak can provide information regarding the defect amount in ZnO samples [55]. In our case, the largest ratio was observed for the No. 3 sample, which indicates the highest number of defects for this compound.

### 2.1. Electrochemical Characterization of SPCE and SPCE/ZnO-Nafion Electrodes

**Figure 5:**
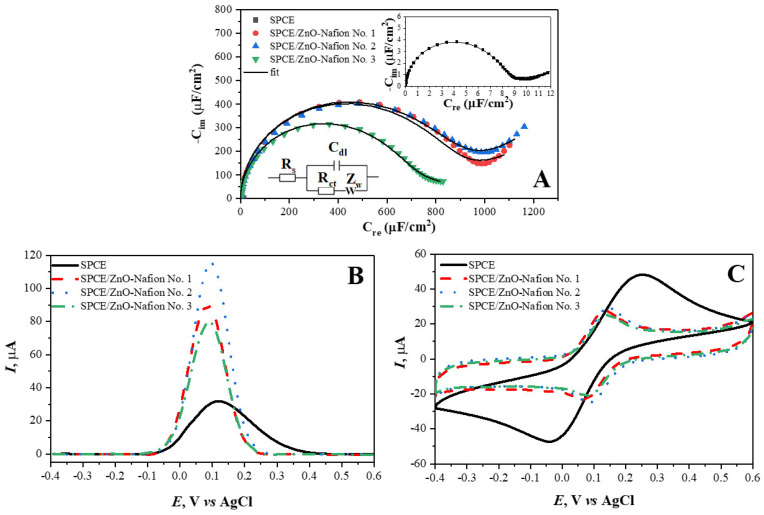
The Cole–Cole plot of EIS spectra (**A**), cyclic voltammograms (**B**), and differential pulse voltammograms (**C**) of SPCE (—), SPCE/ZnO-Nafion No. 1 (- - -), SPCE/ZnO-Nafion No. 2 (· · ·), and SPCE/ZnO-Nafion No. 3 (— ·). Bias potential for EIS 0 V vs. Ag/AgCl, amplitude 10 mV. Potential ranges from −0.4 to +0.6 V, CV scan rate 0.05 V/s, DPV step size of 4 mV, pulse height of 0.05 V, pulse period of 100 ms, pulse width of 50 ms. EIS measurements were performed in 0.01 M PBS, pH 7.4, CV and DPV measurements were performed in 0.01 M PBS, pH 7.4, containing 2.5 mM [Fe(CN_6_)]^3−/4−^.

The different ZnO-Nafion samples were drop-casted onto SPCE and characterized using EIS. The spectra of the different SPCE/ZnO-Nafion samples are shown in Figure 5A. ZnO was synthesized in several ways, yielding ZnO No. 1, ZnO No. 2, and ZnO No. 3. Very often, the EIS spectra are analyzed in the form of Nyquist plots, which analyzes R_im_ vs. R_ct_ [56], but this way of presentation is not always suitable, especially if the sensing signal is generated due to a change in the double layer capacitance; therefore, in this research, the EIS spectra are presented in the Cole–Cole plots (Figure 5A graph: imaginary complex capacitance component C_im_ vs. real complex capacitance component C_re_). These coordinates are well suited to represent the capacitive type of impedance because of the semi-circular part of the EIS spectra, which is proportional to the electrical capacitance. Figure 5A shows changes in the EIS spectra of SPCE before and after drop-casting ZnO-Nafion (SPCE/ZnO-Nafion No. 1–No. 3). Specifically, the complex capacitance semicircle increased by about 100 times, from 7.7 ± 1.1 µF cm^−2^ (before the drop-casting of ZnO-Nafion) to 631 ± 11–817 ± 18 µF cm^−2^ (after the drop-casting of ZnO-Nafion). The increase in the capacitance could lead to CV measurements being less sensitive since CV, among many other factors, depends on (i) the faradaic current resulting from the oxidation/reduction in the redox probe on the electrode surface, (ii) non-faradaic current occurring due to the capacitive contribution (Ic) determined during charging/discharging the double layer, which is represented in an equivalent circuit (Figure 5A, Inset) as a double layer capacitance (C_dl_) that varies when the voltage is swept, and (iii) the diffusion rate of redox species towards the electrode. The capacitive and faradaic currents form the overall registered current, and both currents increase when the potential sweeping rate increases. This effectively restricts the CV technique’s sensitivity since the high capacitive current might interfere with the sensitivity of the faradaic current, which is proportional to the analyte concentration. DPV (Figure 5C) is a more sensitive method for examining the electrochemical behavior of such systems compared to CV because the capacitive current is minimized. However, it should be taken into consideration that an accurate quantitative analysis using the DPV method is difficult when the charge transfer rate for the redox probe oxidation–reduction is slow enough. An example of this is the CV and DPV measurements for unmodified SPCE showing contradicting results with the CV peak oxidation–reduction currents being the highest compared to samples with immobilized ZnO, while the DPV peak current being the lowest. This phenomenon can be explained by the significantly slower charge transfer rate for unmodified SPCE, evident from potential peak separation in the CV results, affecting the measured DPV response. The reduction in the charge transfer rate results in a decrease in the DPV peak current, the increase in the peak half width, and the shift of the peak potential towards more extreme potentials by an activation overpotential (toward the negative for cathodic process and toward the positive for an anodic one) [57,58,59]. These specific effects are also observed in the DPV response for SPCE, making the quantitative analysis of the SPCE response using DPV technique difficult. However, the modification of SPCE with any of the ZnO samples improves the charge transfer rate significantly, removing these limitations for a further quantitative analysis of the DPV response.

### 2.2. Covalent Immobilization of PSA on SPCE/ZnO-Nafion Electrode and Electrochemical Characterization

**Figure 6:**
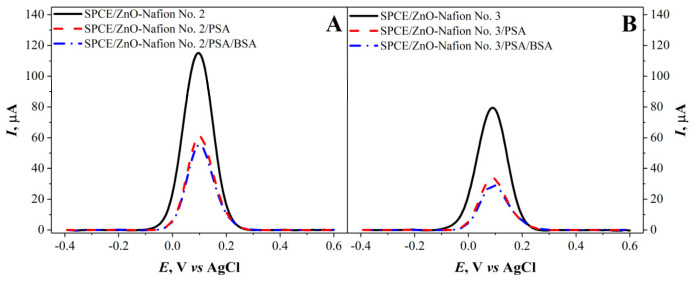
Differential pulse voltammograms of SPCE/ZnO-Nafion No. 2 (**A**), SPCE/ZnO-Nafion No. 3 (**B**) before (—) and after immobilization of PSA (- - -) and blocking the remaining free surface area by BSA (— ·). Potential range from −0.4 to +0.6 V with a step size of 4 mV, pulse height of 0.05 V, pulse period of 100 ms, pulse width of 50 ms in 0.01 M PBS, pH 7.4, containing 2.5 mM [Fe(CN_6_)]^3−/4−^.

As mentioned in the previous section, due to the high capacitive current measured with the CV method for SPCE/ZnO-Nafion, it was decided to use the DPV method to further characterize the electrodes. For SPCE/ZnO-Nafion and SPCE/ZnO-Nafion/PSA, DPV measurements in 0.01 M PBS, pH 7.4, with 2.5 mM [Fe(CN_6_)]^3−/4−^ as a redox probe were carried out and assessed (Figure 6). An increase in the current was seen after the modification of SPCE with ZnO-Nafion. The immobilization of PSA was performed by first exposing the SPCE/ZnO-Nafion-modified electrode to glutaraldehyde vapor and then PSA solution was added, resulting in the cross-linking of the antigen on the modified electrode’s surface. Furthermore, a 2% BSA solution was used to block the remaining free surface to reduce nonspecific interactions during the anti-PSA binding step. The results after the immobilization of PSA exhibited a current drop for all SPCE/ZnO-Nafion samples. The current drop was 52% for SPCE/ZnO-Nafion No. 2 and 63% for SPCE/ZnO-Nafion No. 3 electrodes. In the case of SPCE/ZnO-Nafion No. 1, the immobilization of PSA resulted in a very significant reduction in the registered current, prohibiting the further detection of anti-PSA. As a result, it was decided not to use ZnO-Nafion No. 1 nanostructures for further experiments.

### 2.3. Electrochemical Evaluation of Affinity Interaction of Anti-PSA with PSA Immobilized on the SPCE/ZnO-Nafion Electrode

**Figure 7:**
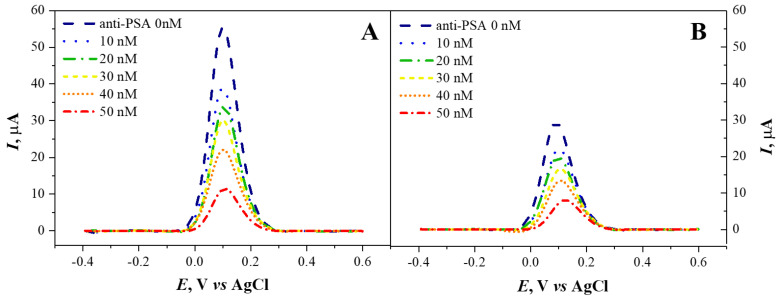
Differential pulse voltammograms of SPCE/ZnO-Nafion No. 2/PSA (**A**) and SPCE/ZnO-Nafion No. 3/PSA (after BSA blocking) (**B**) registered after interaction with different concentrations of anti-PSA. Potential range from −0.4 to +0.6 V with step size of 4 mV, pulse height of 0.05 V, pulse period of 100 ms, pulse width of 50 ms in 0.01 mM PBS, pH 7.4, containing 2.5 mM [Fe(CN_6_)]^3−/4−^.

The interaction of the modified SPCE/ZnO-Nafion/PSA electrodes with monoclonal anti-PSA was tested. For this purpose, immunosensors based on SPCE/ZnO-Nafion No. 2/PSA and SPCE/ZnO-Nafion No. 3/PSA electrodes after treatment with BSA were sequentially incubated in 10 µL of anti-PSA solutions of different concentrations ranging from 10 to 50 nM. Each subsequent incubation was accompanied by DPV measurements (Figure 7) in 0.01 M PBS, pH 7.4, containing 2.5 mM [Fe(CN_6_)]^3−/4−^. There was no significant change registered in the DPV response when the electrode was incubated with PBS buffer containing no anti-PSA; however, a decrease in the recorded peak current was observed when the concentration of anti-PSA was increased. The current decreased from 55.2 ± 1.4 µA at 0 nM of anti-PSA to 11.7 ± 0.3 µA for the solution with 50 nM of anti-PSA for SPCE/ZnO-Nafion No. 2/PSA, and for SPCE/ZnO-Nafion No. 3/PSA, the current decreased from 29.33 ± 0.3 µA for the solution containing 0 nM of anti-PSA to 8.10 ± 0.09 µA for the solution with 50 nM of anti-PSA.

**Figure 8:**
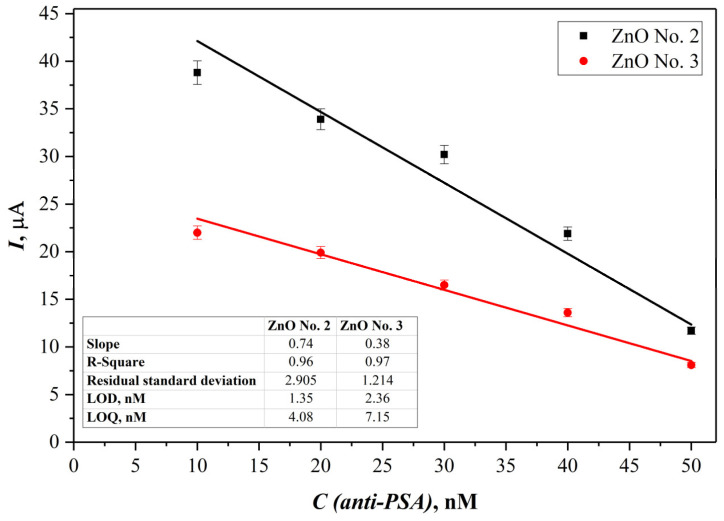
Calibration curves obtained from current SPCE/ZnO-Nafion No. 2 and SPCE/ZnO-Nafion No. 3 peak values, respectively, vs. anti-PSA concentration. Error bars are calculated as a percentage of standard error (*n* = 4).

The linear range, limit of detection (LOD), and limit of quantification (LOQ) for the proposed immunosensor were assessed using the data from the electrochemical experiments carried out, utilizing both SPCE/ZnO-Nafion No. 2/PSA and SPCE/ZnO-Nafion No. 3/PSA methodologies. The calibration curves are displayed in Figure 8.

Immunosensors based both on ZnO No. 2 and ZnO No. 3 showed the same linear range from 10 to 50 nM of anti-PSA; however, the electrode modified with ZnO No. 2 showed a higher sensitivity for anti-PSA detection. The LOD was calculated as 3.3 σ/s and LOQ was calculated as 10σ/s, where σ is the standard deviation for the blank response and s is the slope of the calibration curve. It was revealed that the LOD and LOQ values for the SPCE/ZnO-Nafion No. 2 method were 1.35 nM and 4.08 nM, respectively, while the values calculated from the SPCE/ZnO-Nafion No. 3 data were 2.36 nM and 7.15 nM, respectively. The measurement error was evaluated for ZnO No. 2 and ZnO No. 3, based on immunosensing platforms with the confidence interval of 99.7%, and was ±0.60 nM and ±1.08 nM, respectively.

## 3. Materials and Methods

### 3.1. Materials

Bovine serum albumin (BSA) (≥98%, CAS# 9048-46-8) was obtained from Sigma-Aldrich (Steinheim, Germany). PSA (recombinant human prostate-specific antigen kallikrein-related peptidase 3) (>95%) and mouse monoclonal IgG1 Clone #02 anti-PSA were obtained from SinoBiological Europe GmbH (Eschborn, Germany). K_3_[Fe(CN)_6_] (≥99.0%, CAS# 13746-66-2), K_4_[Fe(CN)_6_] (≥99.0%, CAS# 14459-95-1), and phosphate-buffered saline (PBS) tablets, pH 7.4, were obtained from Sigma-Aldrich (Steinheim, Germany). Zinc acetate dihydrate (Zn(CH_3_COO)_2_∙2H_2_O) (≥99.0%) and ethanol (96%) were received from Sigma Aldrich (XXX), while sodium hydroxide (≥98%), oxalic acid dihydrate (C_2_H_2_O_4_∙2H_2_O) (≥99.5%), and ammonia (NH_3_)_,_ (≥25%) were obtained from Carl Roth (Karlsruhe, Germany). All reagents were of analytical grade and were utilized without additional purification. All aqueous solutions were prepared in deionized water.

### 3.2. Synthesis of ZnO Nanostructures

Three previously reported synthesis methods [46,47,48] were used for the preparation of ZnO nanostructures with different morphology. First, 4.38 g of zinc acetate dihydrate was dissolved in 50 mL of ethanol. Furthermore, a 6 M sodium hydroxide solution was added dropwise to increase the pH value to 8. After stirring this solution for 3 h, a white precipitate was obtained. This mixture was kept ‘to age’ for 12 h and was washed with ethanol and distilled water several times. Finally, after drying and grounding, the powdered material was annealed in a muffle furnace at 400 °C for 4 h with a heating rate of 2 °C/min. The material prepared by this synthesis was labelled as ZnO No. 1.

For the second synthesis, zinc acetate dihydrate and oxalic acid dihydrate were separately dissolved in ethanol and mixed on a magnetic stirrer for 30 min at 45 °C (the molar ratio between Zn^2+^ ions and a complexing agent was 1:2). The solution containing oxalic acid was then slowly added to the zinc acetate solution, and the acquired mixture was continuously stirred for 1 h. The pH value of the mixture obtained was increased to 5 by adding 10% ammonia solution. The resulting solution was left to dry at 80 °C in the furnace for 1 h, and the received powder was heated at 400 °C for 4 h with a heating rate of 2 °C/min. ZnO prepared by this synthetic approach was labelled as ZnO No. 2.

A total of 4.38 g of zinc acetate dihydrate were dissolved in 100 mL of distilled water for the final synthesis. When the clear solution was obtained, 80 mL of 1 M NaOH solution were added dropwise with continuous stirring at 60 °C for 2 h. Additionally, the mixture received was left to age for 12 h. The precipitate was then filtered and washed thoroughly with ethanol and distilled water several times. Last, the acquired powder was dried for 2 h in an 80 °C oven and ground in an agate mortar to receive fine powder. The sample prepared by this method was labelled as ZnO No. 3.

### 3.3. ZnO Characterization Techniques

X-ray diffraction (XRD) analysis was performed with a Miniflex II diffractometer (Rigaku, The Woodlands, TX, USA) using a primary beam Cu Kα radiation (λ = 1.541838 Å) in a 2θ range from 20° to 70° with a step of 0.02° and a scanning speed of 5°/min. The recorded XRD patterns were refined by the Rietveld method using the Fullprof suite. To calculate the crystallite size Scherrer’s equation (D = Kλ/(βcosΘ), where K is the shape factor, λ is the X-ray wavelength, β is the full width at half maximum in radian, and Θ is the Bragg diffraction angle) was used. To correctly determine the β for our samples, the β was measured for the corundum standard.

The Alpha FT-IR spectrometer (Bruker, Ettlingen, Germany) was used for the FT-IR analysis of the compounds. All the spectra were recorded at an ambient temperature in the range of 4000–400 cm^−1^. The morphology of the samples was investigated using a scanning electron microscope (SEM) SU-70 (FE-SEM, Hitachi, Tokyo, Japan). The particle size distribution was estimated from SEM micrographs using ImageJ software version 1.5.3 (Jolla, CA, USA). The emission spectra were recorded using an Edinburgh Instruments FLS980 spectrometer (Edinburgh Instruments Ltd., Kirkton Campus, Livingstone, UK) equipped with a double emission monochromator, a cooled (−20 °C) single-photon counting photomultiplier (Hamamatsu R928P, Iwata, Japan), and a 450 W Xe lamp. The emission spectra were corrected by a correction file obtained from a tungsten incandescent lamp certified by the National Physics Laboratory, UK. The emission spectra were obtained by exciting the samples with 325 nm of light.

### 3.4. Deposition of ZnO Nanostructures on SPCE

**Figure 9:**
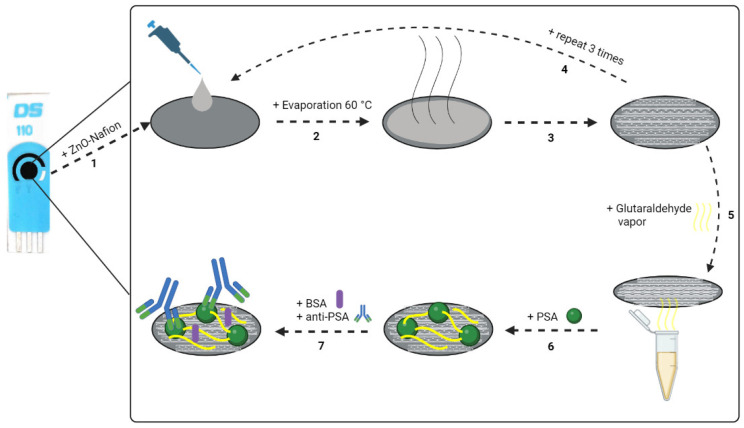
Schematic representation of SPCE modification: 1—ZnO-Nafion deposition; 2,3—evaporation at 60 °C; 4—the drop-casting procedure was repeated 3 times; 5—incubation in glutaraldehyde vapor; 6—immobilization of PSA; 7—blocking of the remaining free surface of the pre-modified electrode with 2% BSA, following the affinity interaction with anti-PSA.

The optimal parameters for ZnO and PSA immobilization were chosen based on previous research [60]. The 2% Nafion mixture was prepared by diluting 10% Nafion solution in ethanol and adding NaOH to increase the pH until it reached a value of 7.0. Then, 2 mg of ZnO powder were added to 1 mL of 2% Nafion mixture (ZnO-Nafion). The final concentration of ZnO in the mixture was 25 mM. Then, 8 µL of the prepared ZnO-Nafion mixture were drop-casted on the working electrode surface of the SPCE (Figure 9, step 1). The modified electrode was dried in the furnace at 60 °C for 30 min (Figure 9, steps 2 and 3). This drop-casting procedure was repeated 3 times (Figure 9, step 4). After ZnO-Nafion deposition (SPCE/ZnO-Nafion), the electrode was rinsed with deionized water and dried under N_2_ flow.

### 3.5. Covalent Immobilization of PSA on the SPCE/ZnO-Nafion and Affinity Interaction with Anti-PSA

After ZnO deposition and drying, the SPCE/ZnO-Nafion electrode was exposed to glutaraldehyde (GA) vapor by placing the electrode over the 50% glutaraldehyde solution and incubating for 15 min (Figure 9, step 5). Afterwards, SPCE/ZnO-Nafion electrode was incubated with 10 µL of 10 µg/mL PSA prepared in PBS, pH 7.4, at 20 °C for 20 min. The PSA immobilization method was based on the adsorption of PSA to the electrode surface and additional cross-linking through the covalent coupling of the protein’s primary amine functional groups and aldehyde groups of the glutaraldehyde (Figure 9, step 6). The remaining free surface of the modified electrode was blocked by incubating in a 2% solution of bovine serum albumin (BSA) for 10 min. Following PSA immobilization, SPCE/ZnO-Nafion/PSA was incubated with 10 µL of monoclonal anti-PSA solution in PBS, pH 7.4, at 20 °C for 10 min (Figure 9, step 6). After each step of incubation, the modified electrode was rinsed with deionized water and used for further electrochemical measurements.

### 3.6. Electrochemical Measurements

The working electrode surface was electrochemically characterized using a potentiostat operated by Wheeler Microfluidics Lab’s DStat-interface software version 1.4.6 (the University of Toronto, ON, Canada). Metrohm DropSens (Oviedo, Spain) provided SPCE with a geometric area of 0.126 cm^2^. The working and counter electrodes were made of carbon, while silver (Ag) was used as a reference electrode. EIS measurements were performed in 0.01 M of phosphate-buffered saline (PBS) solution, pH 7.4. For the registration of the EIS spectra, a perturbation amplitude of 10 mV was applied in the frequency range within 0.1 Hz–100 kHz, at the potential of the 0 V vs. Ag/AgCl. The electrochemical characterization of the working electrode at different modification stages was carried out using DPV and CV methods. DPV and CV electrochemical measurements were performed in 0.01 M of PBS solution, pH 7.4, in the presence of 2.5 mM of [Fe(CN_6_)]^3−/4−^ as a redox probe. DPV experiments were performed in the potential range from −0.4 to +0.6 V vs. Ag/AgCl with a step size of 4 mV, a pulse height of 0.05 V, a pulse period of 100 ms, and a pulse width of 50 ms. Cyclic voltammograms were registered in the potential window from −0.4 to +0.6 V vs. Ag/AgCl at a scan rate of 50 mV/s. All the experiments were carried out at room temperature (20 °C).

## 4. Conclusions

In this study, three different synthesis methods were used for the successful preparation of phase-pure ZnO nanostructures. The morphology of the obtained materials varied from spherical to different sized rod-like particles. The shape and size of the particles influenced both the photoluminescence and electrochemical properties. The position of photoluminescence maxima in the visible region varied in the 620–665 nm interval, and the intensity ratio between the UV peak and visible peak was largest for the No. 3 sample, indicating the largest number of defects. The DPV method was found to be more suitable than CV for the detection of the redox probe since the significant capacitive current resulting from the immobilized ZnO was minimized. The immobilization of all ZnO nanostructures on SPCE resulted in a significant increase in the registered DPV oxidation currents. After PSA immobilization, rod-shaped ZnO No. 2 and ZnO No. 3 modified SPCE were determined to be the more suitable platforms for anti-PSA detection than for spherical ZnO No. 1. When comparing rod-like ZnO nanostructures, the ZnO No. 2 modified SPCE platform was superior, exhibiting 1.75 times lower LOD and LOQ for anti-PSA detection compared to that of ZnO No. 3.

## Figures and Tables

**Table 1:** Unit cell parameters, cell volume, and c/a ratio for different ZnO samples.

	a, Å	c, Å	a/c	V, Å^3^
No. 1	3.2451(9)	5.1990(4)	1.602	47.41(7)
No. 2	3.2480(8)	5.2059(0)	1.603	47.56(4)
No. 3	3.2518(6)	5.2089(0)	1.602	47.70(2)

## Data Availability

Not applicable.
